# Spatiotemporal Aspects of Hendra Virus Infection in Pteropid Bats (Flying-Foxes) in Eastern Australia

**DOI:** 10.1371/journal.pone.0144055

**Published:** 2015-12-01

**Authors:** Hume Field, David Jordan, Daniel Edson, Stephen Morris, Debra Melville, Kerryn Parry-Jones, Alice Broos, Anja Divljan, Lee McMichael, Rodney Davis, Nina Kung, Peter Kirkland, Craig Smith

**Affiliations:** 1 Queensland Centre for Emerging Infectious Diseases, Biosecurity Queensland, Department of Agriculture and Fisheries, Brisbane, Queensland, Australia; 2 Wollongbar Primary Industries Institute, Department of Primary Industries, Wollongbar, New South Wales, Australia; 3 Institute of Wildlife Research, School of Biological Sciences, University of Sydney, Sydney, New South Wales, Australia; 4 Elizabeth Macarthur Agricultural Institute, Department of Primary Industries, Menangle, New South Wales, Australia; 5 EcoHealth Alliance, New York, New York, United States of America; 6 Department of Agriculture, Canberra, Australian Capital Territory, Australia; 7 Australian Museum, Sydney, New South Wales, Australia; 8 School of Veterinary Science, University of Queensland, Gatton, Queensland, Australia; 9 Biosecurity Queensland, Department of Agriculture and Fisheries, Brisbane, Queensland, Australia; Metabiota, UNITED STATES

## Abstract

Hendra virus (HeV) causes highly lethal disease in horses and humans in the eastern Australian states of Queensland (QLD) and New South Wales (NSW), with multiple equine cases now reported on an annual basis. Infection and excretion dynamics in pteropid bats (flying-foxes), the recognised natural reservoir, are incompletely understood. We sought to identify key spatial and temporal factors associated with excretion in flying-foxes over a 2300 km latitudinal gradient from northern QLD to southern NSW which encompassed all known equine case locations. The aim was to strengthen knowledge of Hendra virus ecology in flying-foxes to improve spillover risk prediction and exposure risk mitigation strategies, and thus better protect horses and humans. Monthly pooled urine samples were collected from under roosting flying-foxes over a three-year period and screened for HeV RNA by quantitative RT-PCR. A generalised linear model was employed to investigate spatiotemporal associations with HeV detection in 13,968 samples from 27 roosts. There was a non-linear relationship between mean HeV excretion prevalence and five latitudinal regions, with excretion moderate in northern and central QLD, highest in southern QLD/northern NSW, moderate in central NSW, and negligible in southern NSW. Highest HeV positivity occurred where black or spectacled flying-foxes were present; nil or very low positivity rates occurred in exclusive grey-headed flying-fox roosts. Similarly, little red flying-foxes are evidently not a significant source of virus, as their periodic extreme increase in numbers at some roosts was not associated with any concurrent increase in HeV detection. There was a consistent, strong winter seasonality to excretion in the southern QLD/northern NSW and central NSW regions. This new information allows risk management strategies to be refined and targeted, mindful of the potential for spatial risk profiles to shift over time with changes in flying-fox species distribution.

## Introduction

Hendra virus (HeV) is novel paramyxovirus first described in 1994 in Australia following an outbreak of highly lethal disease in horses and close-contact humans [1–3]. Sporadic equine and human cases continue to occur, with 94 equine and 7 human cases reported to 31 July 2015 [4]. All reported cases have been in the neighbouring eastern Australian states of Queensland and New South Wales. Case fatality rate approaches 90% in horses and 60% in humans [5, 6]. There is currently no established human treatment modality, although a post-exposure monoclonal antibody therapy is in development [7]. An effective vaccine for horses has recently become available [8], however uptake by horse-owners has been limited. Pteropid bats (*Chiroptera*: *Pteropodidae*), commonly known as flying-foxes, are the reservoir of the virus in nature [9–11]. These are nomadic fruit- and blossom-eating bats that forage nocturnally and roost in arboreal colonies by day. There are four mainland Australian species, the black flying-fox (*Pteropus alecto*), the spectacled flying-fox (*P*. *conspiculatus*), the grey-headed flying-fox (*P*. *poliocephalus*) and the little red flying-fox (*P*. *scapulatus*), weighing up to 1 kg, with a wing-span of up to 1.2m. Contemporary colonies generally comprise thousands or tens of thousands of bats, although historically, colonies of hundreds of thousands or millions of bats have been recorded [12–14]. While much is known of the clinical, pathogenic and epidemiological features of HeV infection in horses and humans [5, 6, 15–22], the ecology of infection in flying-foxes is incompletely understood, and its elaboration has been incremental, reflecting the challenges of disease surveillance in a highly mobile wildlife population. Halpin et al [10] identified flying-foxes as the putative natural reservoir of the virus in 1996, two years after the first reported equine cases; Field [11] subsequently elaborated the occurrence and frequency of infection in flying-foxes, and with others, provided initial insights into disease ecology [18, 19, 23–26]; Breed et al [27–29], Plowright et al [30, 31] and Edson et al [32, 33] further explored aspects of disease ecology. Field et al [26] showed that the prevalence of Hendra virus excretion in flying-foxes varied significantly with year, providing a biologically plausible basis for the variable annual case frequency evident in horses. However, several fundamental questions about the infection and excretion dynamics in flying-foxes and the factors associated with spillover from flying-foxes to horses remain unanswered [24, 25, 27, 34].

We sought to identify key spatial and temporal factors associated with HeV excretion in flying-foxes over an extended latitudinal gradient in eastern Australia which encompasses all reported equine case locations. The aim was to strengthen knowledge of Hendra virus ecology in the reservoir host to improve risk prediction and exposure risk mitigation strategies, and better protect horses and humans.

## Methods

### Ethics statement

Fieldwork in all locations and on all occasions was approved under the following permits: in Queensland, the (then) Department of Employment, Economic Development and Innovation Animal Ethics Committee (AEC) Permit SA 2011/12/375 and the Environmental Protection Agency/Department of Environment and Resource Management Scientific Purposes Permits WISP05810609 and WISP14100614; in New South Wales, The University of Sydney AEC Permit 04/3-2011/1/5498, the Elizabeth Macarthur Agricultural Institute AEC Permit M11/15, the Office of Environment and Heritage AEC Permit 120206/02, and the Office of Environment and Heritage Scientific Licences SL100086 and SL 100537. Sample collection entailed collecting urine from underneath roosting flying-foxes. No flying-foxes were handled, captured or sacrificed. Full details of the sample collection methods are presented below. All aspects of the methods were specifically approved under the permits. Sample collection was undertaken by trained and experienced teams.

### Study design and location

A longitudinal survey of flying-fox roosts in the eastern Australian states of Queensland (QLD) and New South Wales (NSW) was undertaken from 3 July 2011 to 14 November 2014. Known, frequently occupied and accessible roosts along a 2300 km transect from northern QLD to southern NSW were selected and enrolled on a staggered entry. Sampling events were conducted at approximately monthly intervals, with variation reflecting negative weather events, the movement/absence of flying-foxes, and logistic issues. For logistical reasons, sampling in QLD commenced earlier (July 2011) and finished earlier (June 2014) than in NSW (April 2012—November 2014).

### Sample collection

Sample collection was largely as described in Edson et al [32]. Briefly, on each sampling occasion, new plastic sheeting (3.6 m x 2.6 m) was placed under trees in which flying-foxes were roosting, usually pre-dusk. Ten sheets were typically used to ensure uniform sampling intensity at each roost regardless of roost size. Data including roost size, species composition, and reproductive status were recorded. The following morning at dawn, pooled urine samples were collected from each sheet using a graduated micropipette and 1 mL filter tip, placed in a graduated screw-cap 2 mL micro cryotube, and held on wet ice. The target sample size and volume on each occasion was 30 x 1.25 mL, with typically three or four pooled samples from each sheet. Pooled samples were methodically collected from discrete sections of each sheet to minimise an individual bat's potential contribution to multiple pools, and typically consisted of 10–20 discrete urine droplets. Rain-affected sampling events were abandoned. After sample collection, 50 μL samples of urine were added to 130 μL of lysis buffer (MagMax Part number AMB500) to inactivate virus particles. In QLD, this process usually occurred in the field, with the processed samples then packed according to IATA-approved protocols and refrigerated, pending overnight shipment to the *Biosecurity Sciences Laboratory* (BSL) in Brisbane. In NSW, the unprocessed pooled samples were similarly packed, and forwarded to the *Elizabeth Macarthur Agricultural Institute* (EMAI) in Menangle, where they were processed within 24 hours of collection. Personal protective equipment during field work typically consisted of overalls or cover shirt and pants, hat with head torch, rubber boots and double nitrile gloves. Equipment was routinely decontaminated with Virkon^™^; waste was similarly decontaminated and bagged and disposed of at BSL or EMAI, or where impractical, at a licensed waste management facility.

### Sample testing

Samples collected in each state were stored and tested in their respective laboratories in accordance with agreed and standardised protocols. Briefly, the samples in lysis buffer underwent total nucleic acid extraction in a Physical Containment Level 3 (PC3) laboratory using the Kingfisher^™^ automated extraction process according to manufacturer’s instructions. Extracts were assayed for Hendra virus RNA using a quantitative RT-PCR targeting the Hendra virus M gene [35]. Samples yielding a Ct value less than 40 were defined as positive.

### Data management and analysis

Roost and sampling event data were recorded on field data sheets, which were subsequently entered into an Excel^™^ spreadsheet, with Hendra virus PCR results added as they became available. Each state maintained its own dataset in a standardised format. On completion of the study, the datasets were investigated for data inconsistencies, cleaned and merged to form a single data set. During the analysis, the number of positive samples of the total number tested was assumed to conform to the binomial probability distribution, which is defined by the true success probability and the number of trials. Exploratory analysis showed that success rates varied over time and by site with some locations indicating seasonal changes in prevalence. The data were thus used to estimate success probability as a function of location and time by fitting a generalised linear model of form:

$$\text{log}\left(\frac{p}{1-p}\right)=Xb+Zu+e$$

The model matrix X defines indicator variables for change in log-odds of success due to sites (location of roost) plus a time gradient at each site. Coefficients are stored in the vector b. The matrix Z contains a cubic spline basis for the series of times at each site with corresponding coefficients stored in the vector u. The Zu component of the model enables a smooth departure from linearity over time for each site as might be driven by seasonality. Coefficients were estimated by application of the ASREML package [36] in the R environment [37]. Predicted detection rates and approximate standard errors were obtained for each site and interpolated to daily observations for the sampling period within each site. These daily estimates were used to assess seasonality of excretion. Comparison of average positive test rates between sites was enabled by predicting average site probabilities over each observation period.

## Results

**Fig 1:**
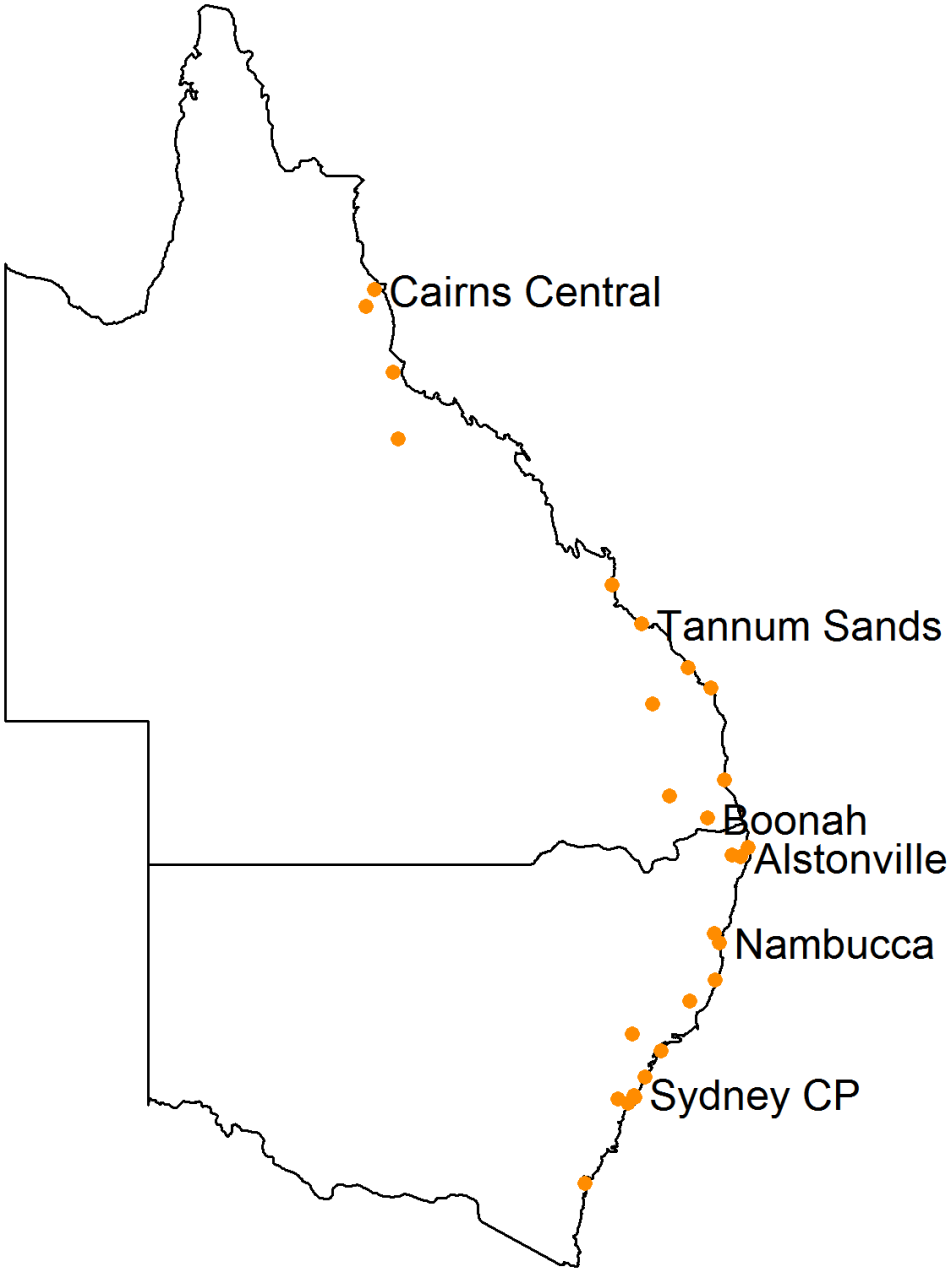
Geographic locations of 27 roosts^1^ surveyed for Hendra virus in flying-fox pooled urine samples. (Sydney CP = Sydney Centennial Park). ^1^ Roosts with five or more sampling events.

A total of 14,988 pooled urine samples were collected from 50 roosts across 20° of latitude from Cairns in northern QLD (latitude 16.9° S) to Bateman’s Bay in southern NSW (latitude 35.7° S), spanning maritime temperate, subtropical and tropical climates of eastern Australia. Roosts with less than five sampling events (S1 Table) were excluded to facilitate the longitudinal analysis, leaving a total 13,968 samples (QLD n = 6,801; NSW n = 8,187) from 27 roosts (Fig 1 and S1 Fig)

**Fig 2:**
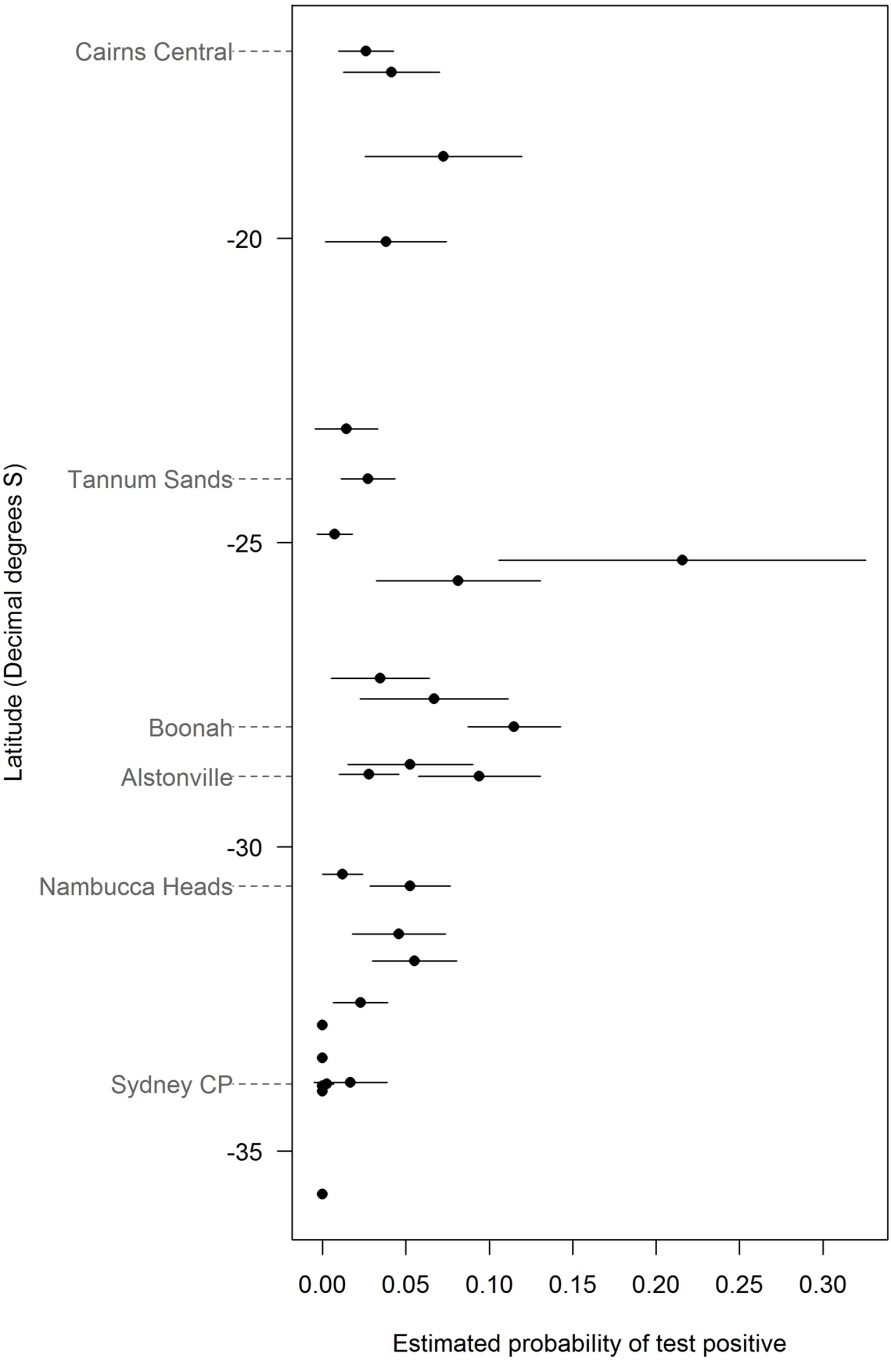
Spatial distribution of estimated probability of positive tests (and 95% CI) averaged over the period of observation for each roost, arranged according to latitude (Sydney CP = Sydney Centennial Park).

The analysis first estimated a mean probability of HeV detection (“positivity”) across all time periods for each roost (Fig 2). HeV positivity was minimal in roosts south of Newcastle (0.0 to 0.02), and generally increased north from Newcastle to the NSW—QLD border region (e.g. Alstonville 0.10, Boonah 0.12). Further north, mean positivity decreased but remained moderate (e.g. Tannum Sands 0.03, Cairns 0.03). The highest mean probability of detection was at the Hervey Bay roost (0.21), however, this is accompanied by large uncertainty arising from the small number of observations collected in a short time interval (S1 Fig).

**Fig 3:**
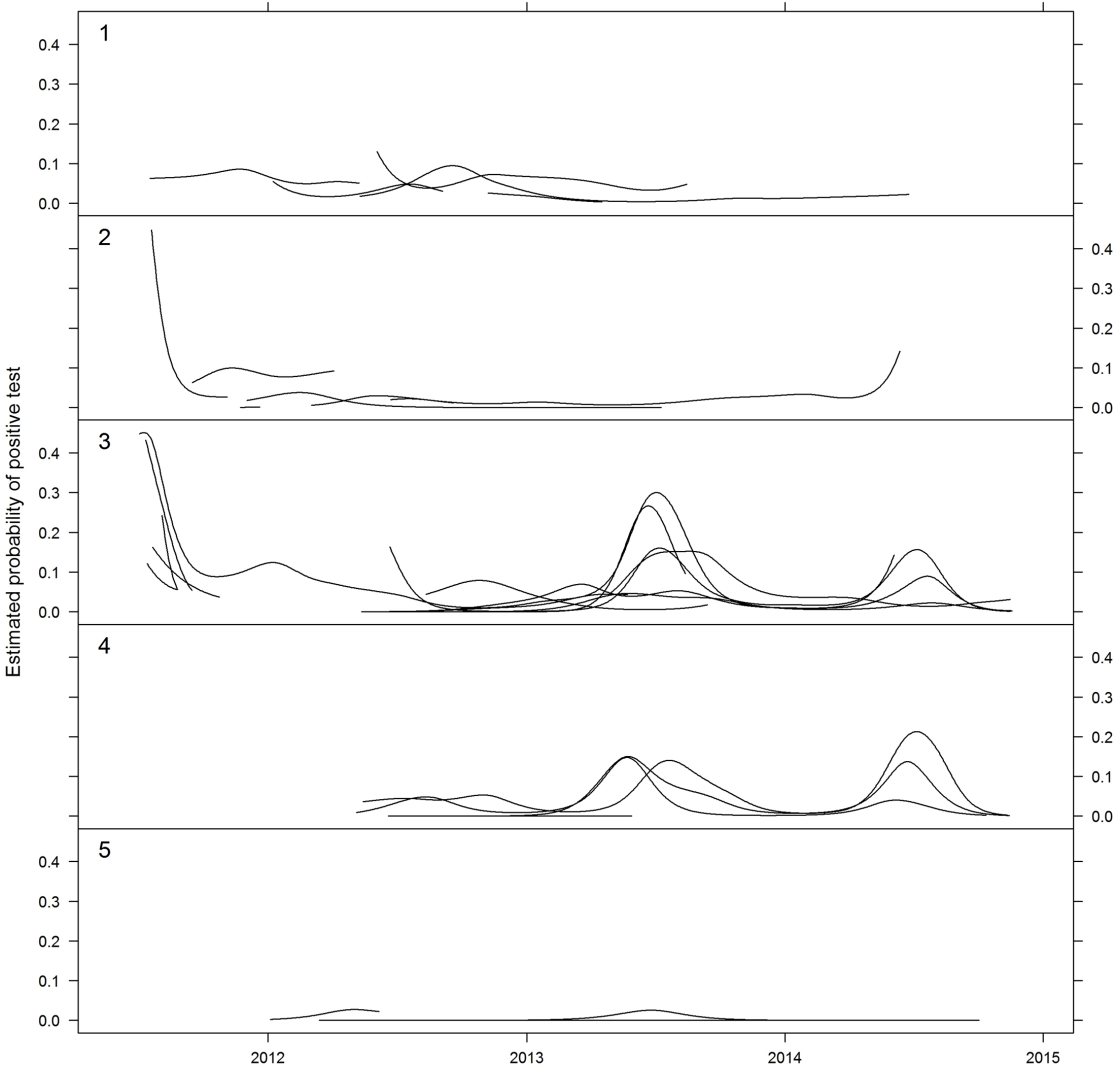
Estimated time trends in positivity of flying-fox urine for Hendra virus in five contiguous coastal regions of eastern Australia. The regions were: 1. northern QLD (Cairns to Charters Towers), 2. central QLD (Yeppoon to Gayndah), 3. southern QLD/northern NSW (Redcliffe to Nambucca Heads), 4. central NSW (Port Macquarie to Newcastle), and 5. southern NSW (Avoca to Batemans Bay).

Seasonality of excretion was evident from the model estimates. Fig 3 shows the time-trends in the probability of HeV detection, with roosts grouped into five contiguous coastal regions reflecting statistically significant latitudinal spatial clustering. There was a strong tendency for mid-year (winter) peaks in HeV excretion in regions 3 and 4. This seasonality was much less evident in the more northern roosts in regions 1 and 2.

**Fig 4:**
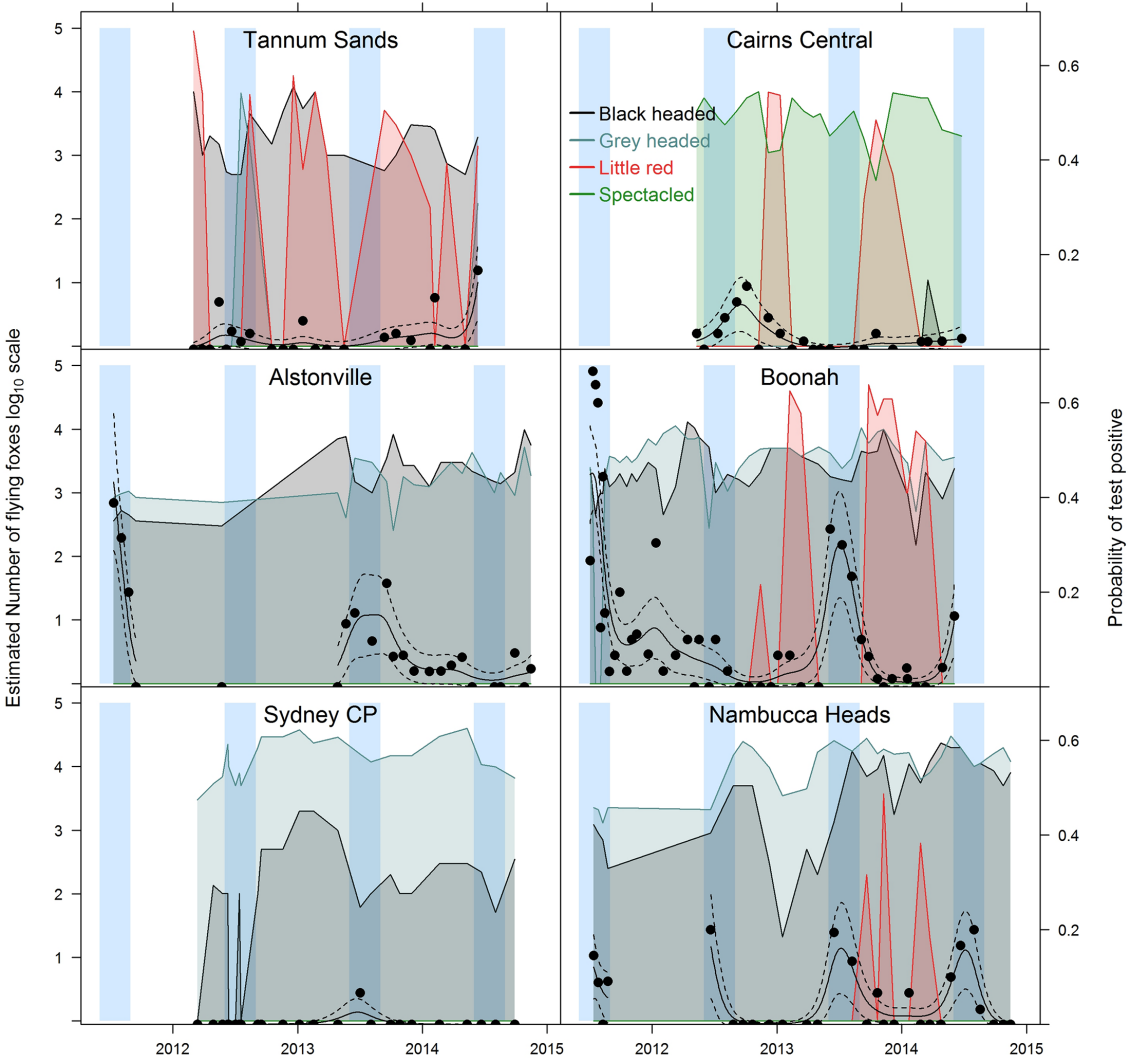
Observed (points) and estimated (smooth curves) probability of Hendra virus being detected in flying-fox pooled urine samples collected at selected roosts over the duration of study. Dashed lines indicate the predicted probability plus or minus two standard errors. Vertical shaded regions show winter periods. Shaded polygons show estimated (observed) species count (key lower right) over time on the log_10_ scale, where 1 log_10_ represents 10 individuals and 5 log_10_ represents 100,000 individuals. (Sydney CP = Sydney Centennial Park).

Data on flying-fox species and numbers for six of the most extensively sampled roosts are shown in Fig 4, accompanied by the proportion of HeV-positive samples at each time point. These six roosts are spaced along the north-south axis of the study area and serve as representative examples for the remaining 21 roosts (data for all 27 roosts is given in S1 Fig). The data clearly show how the four species of flying-fox were distributed throughout the study area and with time. The plots demonstrate that in the south (e.g. Sydney), grey-headed flying-foxes consistently outnumbered black flying-foxes 100 to 1 (2 logs) or more, and HeV detection was zero or very low. Moving north (e.g. Nambucca Heads to Alstonville to Boonah), black and grey-headed flying-foxes occurred in approximately equal proportions, HeV positivity was relatively high, and detection was winter dominant. Further north into central QLD (e.g. Tannum Sands), where black and little-red flying-foxes predominate, HeV positivity is lower and the level of detection more stable, with less evidence of seasonality. A similar pattern is evident in northern QLD (e.g. Cairns), where spectacled flying-foxes are the predominant species. Within the individual roosts (S1 Fig), the regional temporal trends illustrated in Fig 3 are evident, but there is a lack of any consistent association between the temporal peaks of HeV excretion and the presence or absence of specific flying-fox species.

## Discussion

The impetus for this work followed an unprecedented wide-area outbreak of HeV infection in horses in 2011 that spanned the eastern Australian states of Queensland and New South Wales. There were more separate incidents (18, involving 23 cases) reported in a 12 week period between late June and early September 2011 than in the preceding 16 years since HeV was first described [23]. The surveillance reported here was commissioned by a joint state and national government task force in response, to better understand the epidemiology and ecology of HeV infection in Australian flying-foxes, and so inform spillover risk management strategies. The scale of this study is exceptional in terms of its geographic expanse (20° of latitude), duration of surveillance (three years) and sample size (almost 14,000). A sampling methodology using pooled urine samples collected under roosting flying-foxes, previously shown to be an effective tool in population-level surveillance [26, 32, 38], offered the only logistically feasible means to conduct surveillance on this scale. The study makes key findings about HeV excretion prevalence over space and time, about seasonality of excretion, and about the reservoir host status of various flying-fox species, and in doing so, facilitates revision of previous and current hypotheses regarding spillover risk.

The data indicate that on average there were higher levels of virus excretion in roosts in southern QLD/northern NSW, moderate levels of excretion in roosts in central and northern QLD and central NSW, and low or zero excretion in the roosts in southern NSW. This spatial variation in HeV excretion levels is more consistent with species distribution than with a simple latitudinal gradient, with the moderate and higher levels of excretion paralleling the presence of black flying-foxes (and in northern QLD, the paraphyletic spectacled flying-fox [39]), and the zero or low levels in the south paralleling the absence or relative absence of black flying-foxes. The highest HeV positivity rates occurred where black or spectacled flying-foxes were present; conversely, a nil or very low positivity rate occurred in exclusive grey-headed flying-fox roosts, notwithstanding their demonstrated connectivity with roosts containing black flying-foxes [40–42]. Similarly, the data indicate that little red flying-foxes are not a significant source of virus, as their periodic extreme increase in numbers at some roosts (e.g. Tannum Sands and Cairns, Fig 4) are not associated with any concurrent increase in HeV detection. Indeed, the presence of large numbers of little red flying-foxes would likely ‘swamp’ under-roost pooled urine samples, and result in underestimation of HeV prevalence in co-roosting black flying-foxes (e.g. Boonah, Feb 2014). However, the pooled urine sampling methodology did not allow us to specifically ascribe detections in mixed species roost to one or other species, and constrains us from definitively attributing temporal peaks in HeV excretion to a particular species. While flying-fox species and latitude are heavily confounded, the empirical evidence in this large study is consistent with the findings of several recent studies which indicate that black and spectacled flying-foxes are the primary reservoir host species for HeV [32, 33, 43, 44].

Between-year variation in under-roost HeV detection prevalence has previously been reported [26], and is again evident in multiple roosts in this study. Notwithstanding the potential influence of other factors, the observed spatiotemporal variation in the occurrence of equine cases [4, 22, 24] is consistent with this scenario. Given the typically dynamic nature of flying-fox roosts, between-year variability in HeV infection and excretion more likely reflects the proportion of susceptible individuals at a regional level, and thus the ability to sustain infection in roosts in the region, rather than the presence or absence of virus per se, or roost size per se. Plowright et al [31] have previously argued that a metapopulation model best describes Australian flying-fox populations, and described the effect of this model on HeV transmission dynamics. Our findings of a variable seasonal pattern of excretion in some regions further convey the spatiotemporal complexity of the system. Excluding the southern NSW region because of the very limited HeV positivity, the remaining regions show a similar background of ‘endemic’ infection (consistent with the findings of Breed et al [27]), which, in the southern QLD/northern NSW and central NSW regions is overlain with a strong winter epidemic pattern. While an earlier study by Field et al [26] did not show statistically significant seasonality of excretion, a more recent study by Edson et al (in preparation), which screened individual wild-caught black flying-foxes in south-east QLD in 2013–14, also showed a strong winter pattern of excretion, consistent with our findings. From an epidemiological perspective, an epidemic pattern of infection suggests either more susceptible individuals and/or more effective transmission. While the latter is consistent with our earlier contention that HeV transmission is more efficient in black flying-foxes, the more endemic pattern in central and north QLD where black and spectacled flying-foxes are respectively present suggests a different disease ecology. It is possible that the lack of epidemic overlay in these regions simply reflects a lower prevalence of infection, a lower population density, or other ecological factor(s) which dampens epidemic transmission. However, the winter peaks evident in central Queensland in 2011 and 2014, and in northern Queensland in 2012 suggest that the factors causing the winter epidemic pattern in the south can occur further north, but less frequently and with less intensity. Seasonality of detection of the related Nipah virus has also been reported in flying-foxes in Thailand [45]. The contrasting summer seasonality in that (northern hemisphere) study further supports the likelihood that biological or epidemiological factors underlie henipavirus excretion prevalence in flying-foxes.

Our findings allow a number of prevailing hypotheses in relation to Hendra virus spillover to be reviewed. Several hypotheses pose an association with elements of the strongly seasonal reproductive cycle of flying-foxes, either gestation [11, 27, 30, 34], birthing [46], and/or lactation [27, 30]. Inherent to these hypotheses is the concept of either recrudescence, or increased susceptibility, or increased exposure to infection at this time. The winter spike in excretion seen in southern QLD/northern NSW and central NSW correlates temporally with mid-to-late gestation in black, spectacled and grey-headed flying-foxes, but not with birthing or lactation [12]. However, the absence of a consistent winter spike in central and northern QLD, given the same mid-late gestation timing, is inconsistent with a simple causal association. Another hypothesis suggests an association with the loss of maternal antibodies in the previous year’s birth cohort [31]. Using October as the peak birthing month [12] and 7.5 months as the mean length of maternal antibody decay in black flying-foxes [47], the mid-point of our observed winter excretion spike lags the peak surge in susceptible individuals into the population by 2.5 months. Indeed, it is likely that the peak surge occurs considerably earlier than this, as the terminal waning maternal antibody titres detected by Epstein et al 2013 [47] would unlikely be functionally protective [48]. Thus while the hypothesis is epidemiologically attractive, the lag period is problematic. Further, the same surge of susceptible individuals could be expected to occur at the same time in the central and northern QLD, yet again no consistent winter spike in excretion was seen in these regions. Scanlan et al [49] suggest that extended virus survival time in the environment at lower temperatures may contribute to the observed winter clustering of equine cases seen in southern QLD and northern NSW. How lower environmental temperatures might promote HeV infection and excretion in flying-foxes is less intuitive. However, a recent study by Hawley and Altizer [50] suggests a possible mechanism by which the winter temperature differential might explain the contrast between the excretion pattern seen in central NSW and southern QLD/northern NSW, and that seen further north. Broadly, Hawley and Altizer [50] discuss the potential cost of winter thermoregulation in relation to likely reduced immune system function. This scenario warrants consideration with respect to black flying-foxes, given the relatively recent and rapid southern range shift of this historically tropical species into the subtropical and temperate regions of southern QLD and northern NSW [51].

There are several limitations to the study. Firstly, we recognise the constraints to interpretation inherent with a pooled (urine) sample approach, however the magnitude and consistency of changes in HeV prevalence identified indicate that the methodology is sufficiently robust for the objectives of this study. Additionally, a recent study identifying urine as the primary route of HeV excretion in flying-foxes [33] supports our sampling of urine. Secondly, we assume the sampled roosts are representative of their geographic region, and believe that the dynamic nature of flying-fox roosts supports this assumption. We acknowledge that individual roosts may be over-represented or under-represented in the data, and have thus used an analytical approach that robustly accounts for sample size. As indicated above, species and latitude are heavily confounded, which unavoidably constrained our ability to fit statistical models to characterise more precisely how these variables influence HeV excretion prevalence. Finally, while we have recorded three successive years of marked seasonal variation in HeV excretion in the southern QLD/northern NSW and central NSW regions, we cannot exclude the possibility that this variation was an aberration of those particular years and not reflective of the long term pattern of excretion. Despite these potential limitations, the size and scope of the dataset is unsurpassed, and the findings provide new information to support HeV exposure risk assessment and the development of risk mitigation strategies.

The study findings have substantial ramifications for the mitigation of equine and thus human cases of HeV infection. While not discounting the need for awareness of Hendra virus in the horse industry generally, a risk communication approach that targets horse-owners in central and northern NSW, and southern, central and northern QLD could strategically elevate awareness in these higher risk regions. Similarly, there is the potential to refine the temporal focus of control measures, particularly with respect to the timing of equine vaccinations in the southern QLD/northern NSW and central NSW regions, with a strategic autumn campaign to promote equine HeV vaccination. This would precede the observed regional high-risk winter period of excretion in flying-foxes in these regions, which parallels the respective historical clustering of equine cases. Similarly, the general measures for the mitigation of flying-fox—equine contact could receive a winter emphasis in these regions.

## Conclusion

The study has advanced understanding of HeV infection dynamics in flying-foxes and thereby, understanding of the fundamental drivers for HeV spillover to horses, and indirectly humans. Broadly, the findings convey the variable nature of HeV excretion by flying-foxes in eastern Australia over space and time. Firstly, they show a non-linear relationship between mean HeV excretion prevalence and latitude, with excretion prevalence highest in southern QLD and northern NSW. Secondly, they demonstrate a consistent, strong winter peaking of excretion in southern QLD and central and northern NSW. Thirdly, they provide supporting evidence that black and spectacled flying-foxes are more likely to be associated with HeV excretion than other species. These findings are consistent with the observed spatiotemporal pattern of infection in horses, and demonstrate that HeV infection prevalence in flying-foxes is a fundamental determinant of infection in horses. From a spillover risk management perspective, the new information allows risk management strategies to be refined and targeted. These strategies need to be mindful of the potential for spatial risk profiles to shift over time with changes in flying-fox species distribution.

## Supporting Information

S1 Fig Data from 27 roosts with five or more sampling events showing observed (points) and estimated (smooth curves) probability of test positive for HeV in flying-fox pooled urine samples collected over the duration of study. Dashed lines indicate the predicted probability plus or minus two standard errors. Vertical shaded regions show winter periods. Shaded polygons show estimated (observed) species count (key lower right) over time on the log_10_ scale, where 1 log_10_ represents 10 individuals and 5 log_10_ represents 100,000 individuals. (Sydney RBG = Sydney Royal Botanic Gardens; Sydney CP = Sydney Centennial Park).
(TIF)

S1 Table Location, sample collection dates, and proportion of HeV-positive flying-fox pooled urine samples (in parentheses) for roosts with less than five sampling events. These roosts were excluded from the longitudinal analysis.
(DOCX)
