# Supplementary figures and images for: Spatiotemporal Aspects of Hendra Virus Infection in Pteropid Bats (Flying-Foxes) in Eastern Australia
Source: PLoS One. 2015 Dec 1;10(12):e0144055. doi: 10.1371/journal.pone.0144055 (PMC4666458; doi:10.1371/journal.pone.0144055)

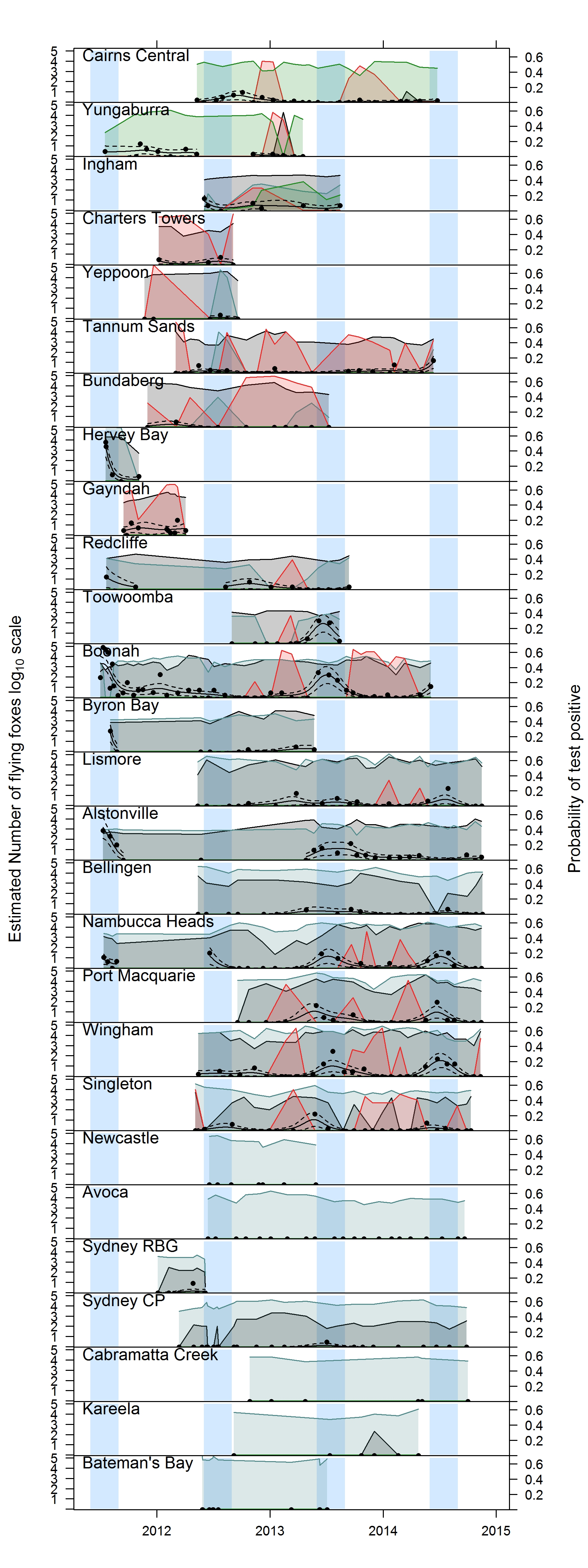

Supplement: S1 Fig — Dashed lines indicate the predicted probability plus or minus two standard errors. Vertical shaded regions show winter periods. Shaded polygons show estimated (observed) species count (key lower right) over time on the log10 scale, where 1 log10 represents 10 individuals and 5 log10 represents 100,000 individuals. (Sydney RBG = Sydney Royal Botanic Gardens; Sydney CP = Sydney Centennial Park). (TIF) [file pone.0144055.s001.tif]
