# Supplementary material for: Spatiotemporal Aspects of Hendra Virus Infection in Pteropid Bats (Flying-Foxes) in Eastern Australia
Source: PLoS One. 2015 Dec 1;10(12):e0144055. doi: 10.1371/journal.pone.0144055 (PMC4666458; doi:10.1371/journal.pone.0144055)
Supplement: S1 Table — These roosts were excluded from the longitudinal analysis. (DOCX) [file pone.0144055.s002.docx]

| **Roost location** | **Sample collection dates (proportion positive)** |
| --- | --- |
| Port Douglas, QLD | 11Sep12(0.00), 03Oct12(0.00) |
| Tolga Scrub, QLD | 18Jul11(0.00), 19Jul11(0.00), 03Jul13(0.27) |
| Townsville, QLD | 01Dec11(0.00) |
| Collinsville, QLD | 22Oct11(0.03) |
| Mt Isa, QLD | 20Oct11(0.00), 15Nov11(0.00) |
| Seaforth, QLD | 03Jul12(0.05) |
| Great Keppel Island, QLD | 28Feb12(0.00) |
| Barcaldine, QLD | 01Sep11(0.00) |
| Jericho, QLD | 10Jan12(0.00) |
| Duaringa, QLD | 03May12(0.00), 07Jun12(0.00), 18Oct12(0.00) |
| Bargara, QLD | 30Nov11(0.00) |
| Coalstoun Lakes, QLD | 04Apr12(0.00) |
| Dalby, QLD | 28Jul11(0.31), 27Apr12(0.03) |
| Sandgate, QLD | 20Jul11(0.22) |
| Regents Park, QLD | 08Jul11(0.00) |
| Carrara, QLD | 26Aug11(0.48), 13Sep11(0.17), 28Sep11(0.06) |
| Canungra, QLD | 05Jul11(0.50), 11Nov11(0.00), 02Nov12(0.00) |
| Warwick, QLD | 05Mar13(0.04) |
| Tocal, NSW | 06May12(0.00) |
| Shellharbour, NSW | 11Sep12(0.00), 19Oct12(0.00), 21Aug13(0.00) |
| Bomaderry, NSW | 14Feb13(0.00), 12Mar13(0.00) |
